# Supplementary figures and images for: A universal method for the purification of C2H2 zinc finger arrays
Source: PLoS One. 2025 Feb 4;20(2):e0318295. doi: 10.1371/journal.pone.0318295 (PMC11793764; doi:10.1371/journal.pone.0318295)

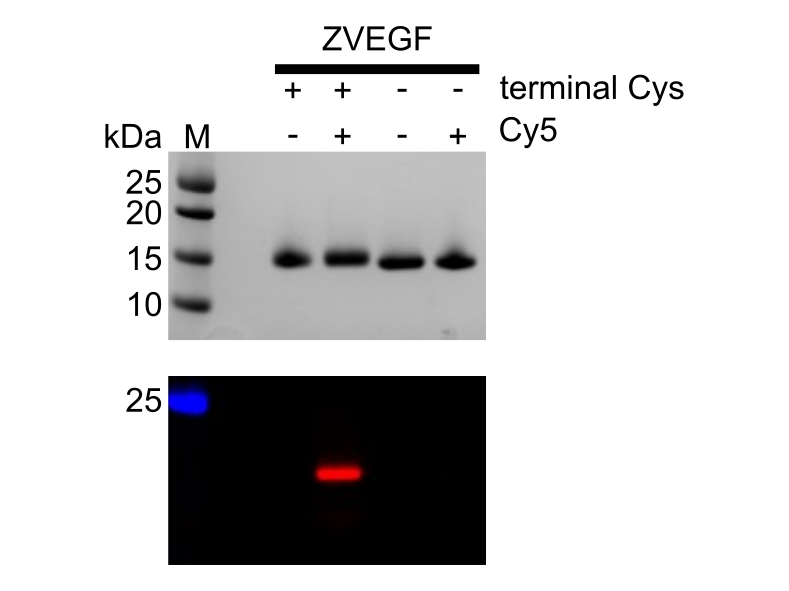

Supplement: S1 Fig — ZVEGF with (+) and without (-) free cysteine were labeled with Cy5and compared by SDS-PAGE. (TIFF) [file pone.0318295.s001.tiff]

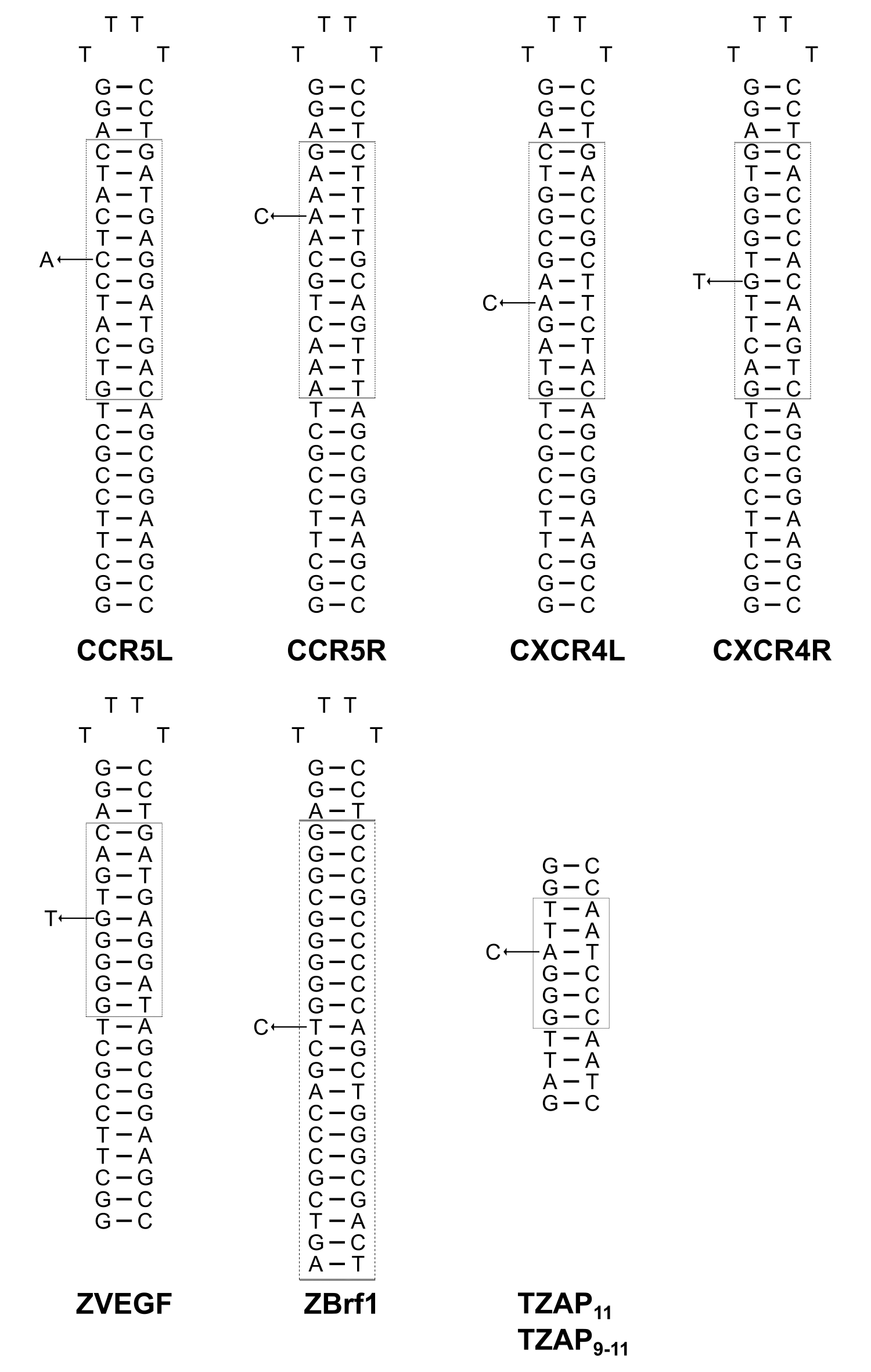

Supplement: S2 Fig — Black boxes indicate the binding site for ZF proteins, and black arrows indicate mutated sites. (TIFF) [file pone.0318295.s002.tiff]

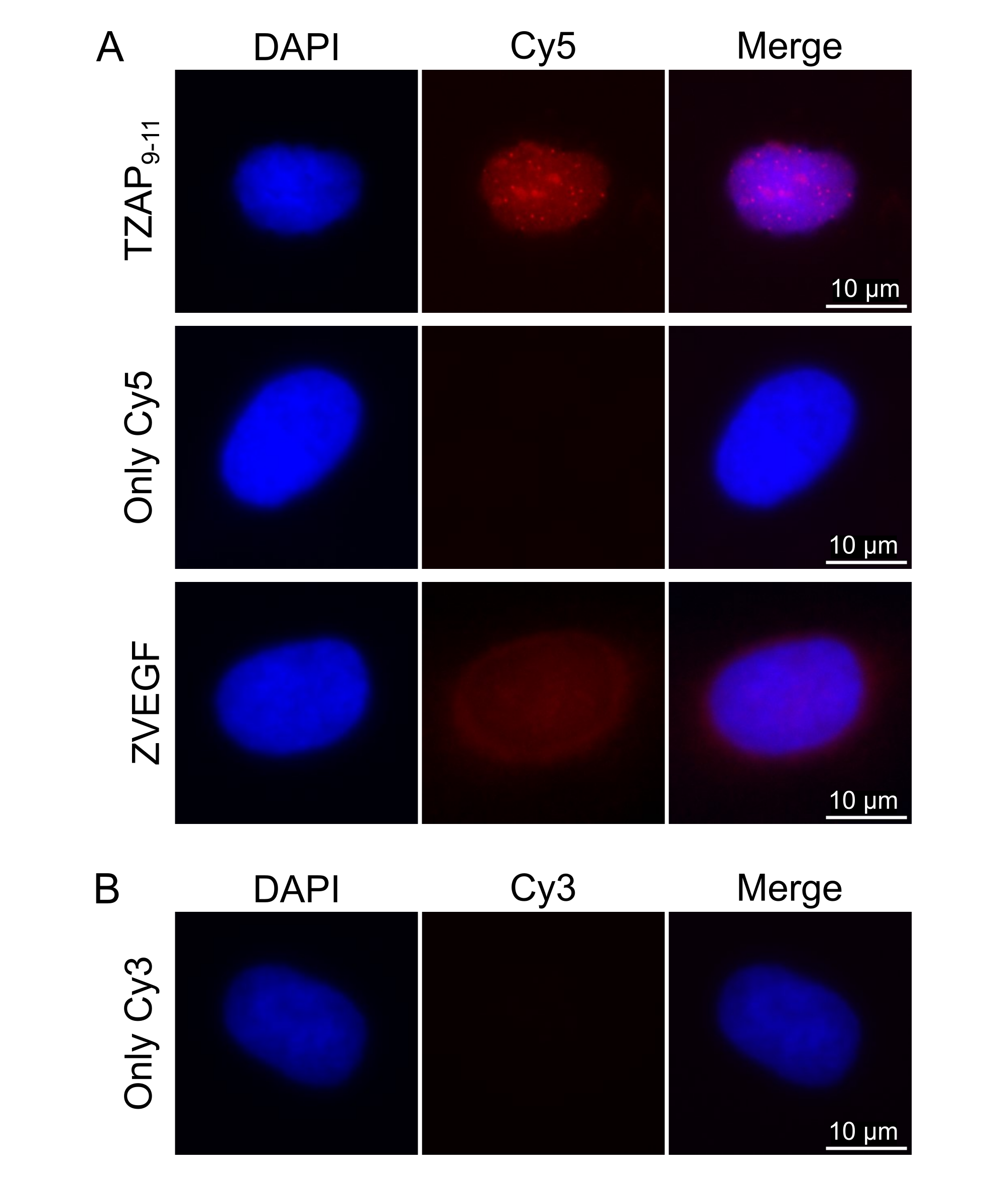

Supplement: S3 Fig — A. Top: Fixed U2OS cells stained with DAPI (blue) and labeled with Cy5-TZAP9–11 probe (red) were imaged on a widefield microscope. Middle: Fixed U2OS cells stained with DAPI (blue) and labeled with Cy5 (quenched with β-ME, red) were imaged on a widefield microscope. Bottom: Fixed U2OS cells stained with DAPI (blue) and labeled with Cy5-ZVEGF (red) were imaged on a widefield microscope. B. Fixed U2OS cells stained with DAPI (blue) and labeled with Cy3 (quenched with β-ME) were imaged on a widefield microscope. Scale bar: 10 μm. (TIFF) [file pone.0318295.s003.tiff]
