# Supplementary material for: A universal method for the purification of C2H2 zinc finger arrays
Source: PLoS One. 2025 Feb 4;20(2):e0318295. doi: 10.1371/journal.pone.0318295 (PMC11793764; doi:10.1371/journal.pone.0318295)
Supplement: S4 Fig — (PDF) [file pone.0318295.s004.pdf]

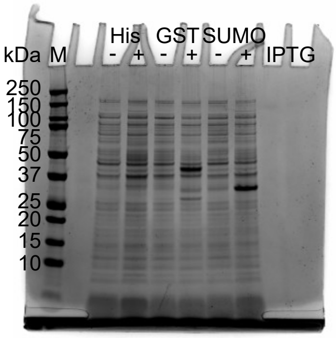

Fig 1A

ChemiDoc™ MP Imaging System: SDS-PAGE

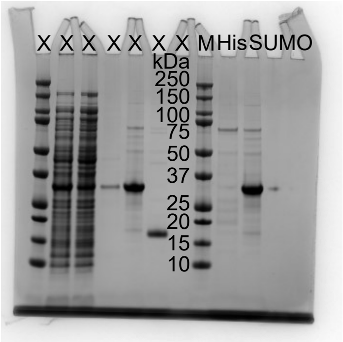

Fig 1B SDS-PAGE

ChemiDoc™ MP Imaging System: SDS-PAGE

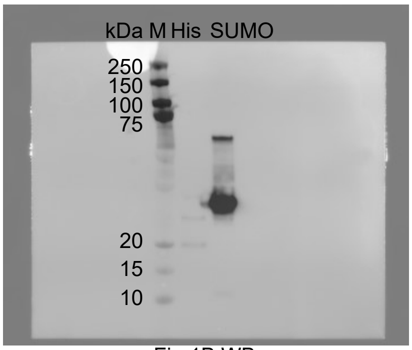

Fig 1B WB

ChemiDoc™ MP Imaging System: western blot

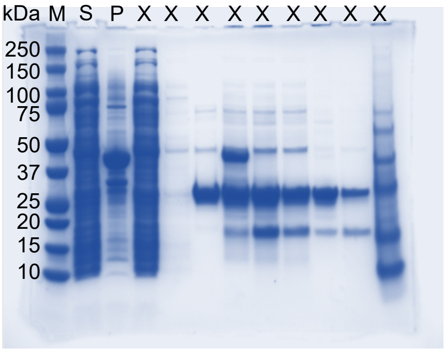

Fig 1C SDS-PAGE

Amersham Imager 600

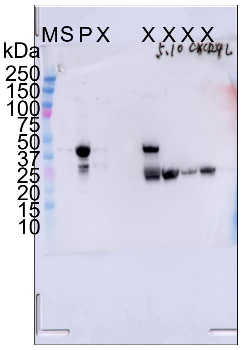

Fig 1C WB

Amersham Imager 600

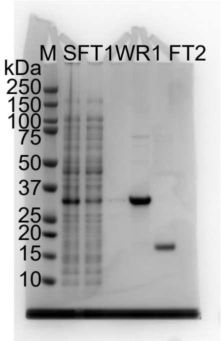

Fig 1E

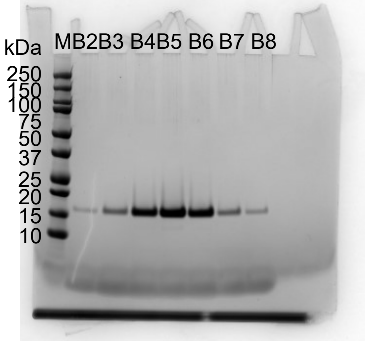

Fig 1F

ChemiDoc™ MP Imaging System: SDS-PAGE

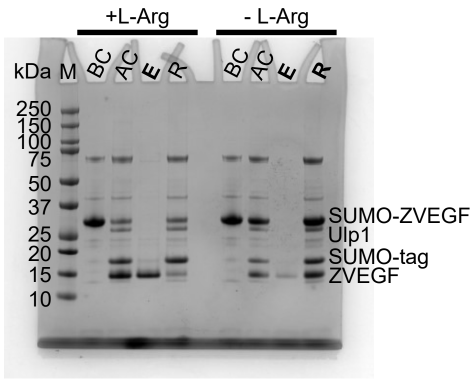

Fig 2

ChemiDoc™ MP Imaging System: SDS-PAGE

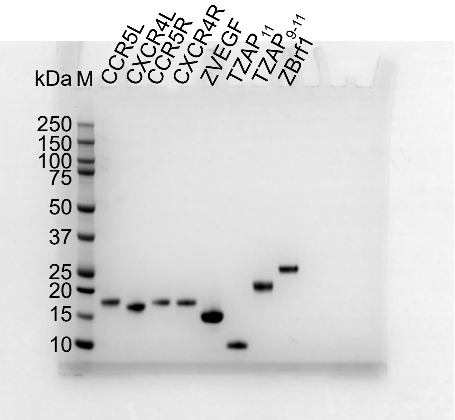

Fig 3A top

ChemiDoc™ MP Imaging System: SDS-PAGE

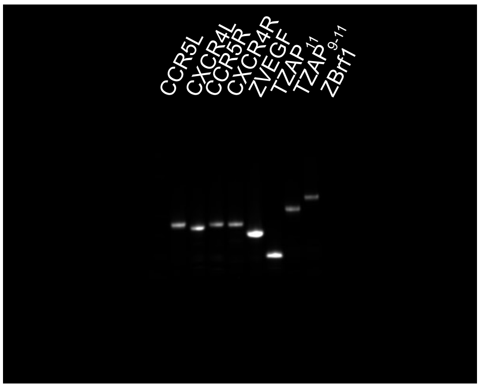

Fig 3A bottom

ChemiDoc™ MP Imaging System: SDS-PAGE

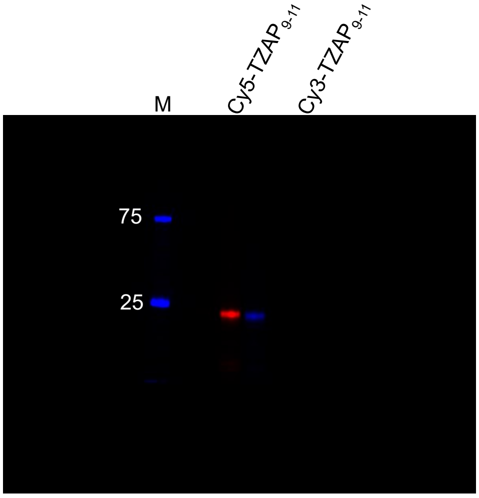

Fig 3B

ChemiDoc™ MP Imaging System: SDS-PAGE

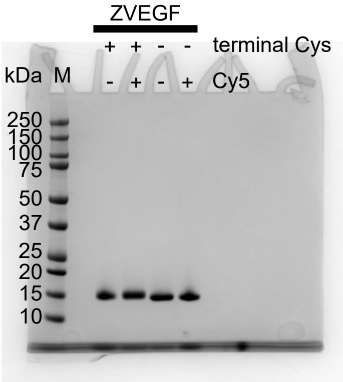

S1 Fig Top

ChemiDoc™ MP Imaging System: SDS-PAGE

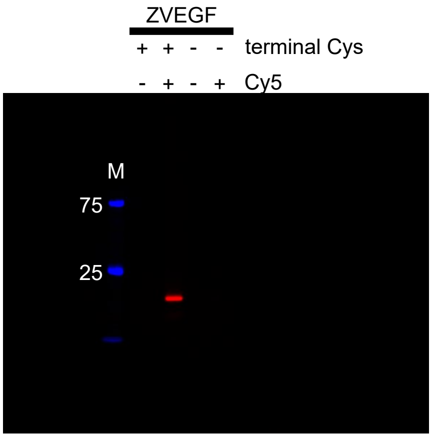

S1 Fig bottom

ChemiDoc™ MP Imaging System: SDS-PAGE

DAPI

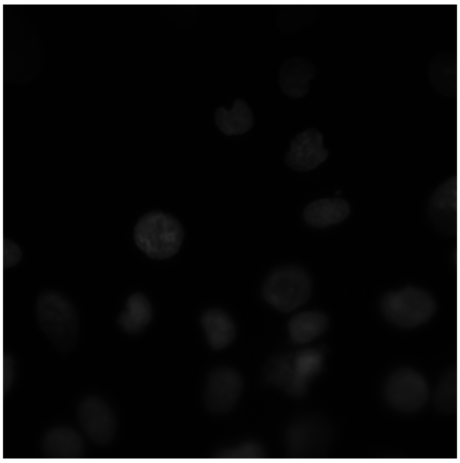

Cy3-TZAP$_{9-11}$

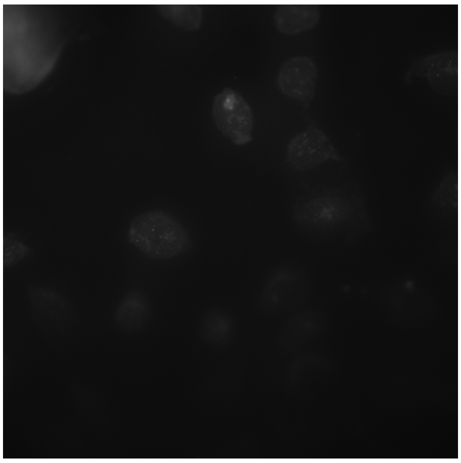

Alexa Fluor 647-TRF2 antibody

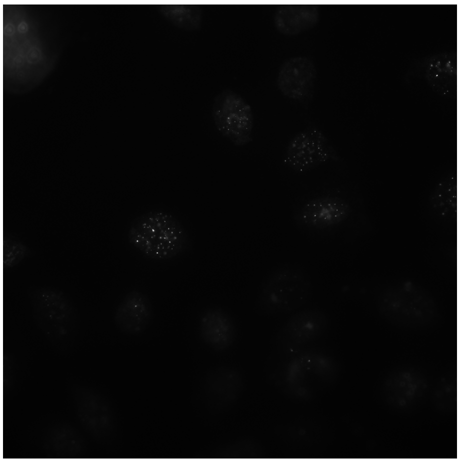

Fig 5
Nikon Microscope Eclipse 80i

DAPI

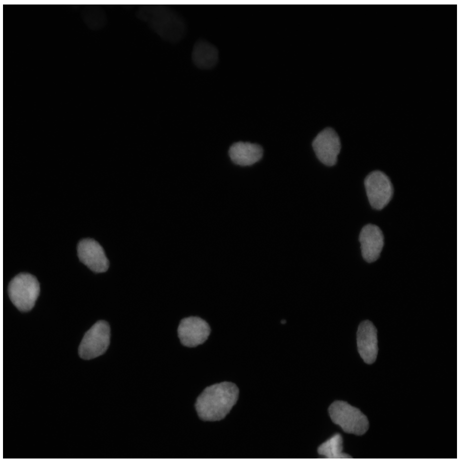

S3A Fig Top
Nikon Microscope Eclipse 80i

Cy5-TZAP$_{9-11}$

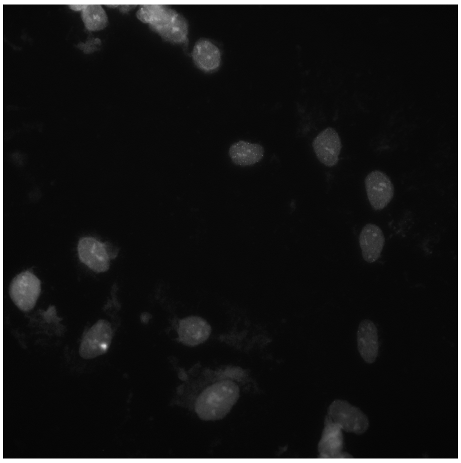

S3A Fig Top
Nikon Microscope Eclipse 80i

DAPI

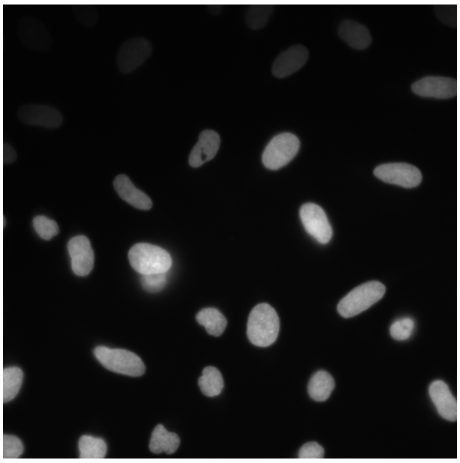

S3A Fig Middle
Nikon Microscope Eclipse 80i

Only Cy5

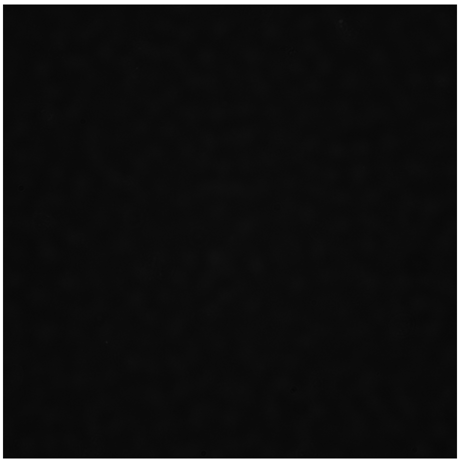

S3A Fig Middle
Nikon Microscope Eclipse 80i

DAPI

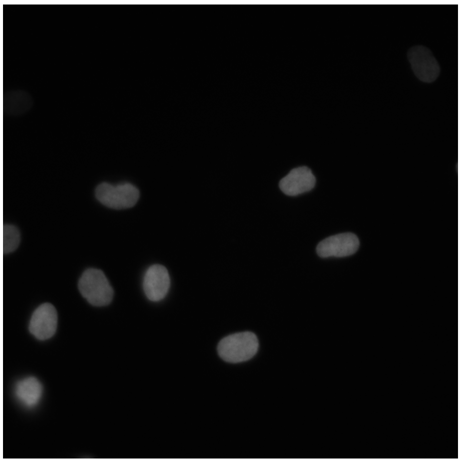

S3A Fig Bottom
Nikon Microscope Eclipse 80i

Cy5-ZVEGF

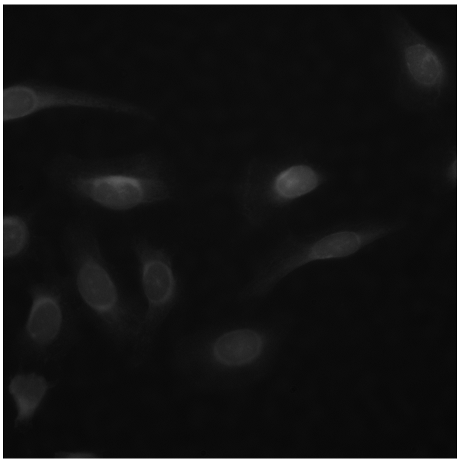

S3A Fig Bottom
Nikon Microscope Eclipse 80i

DAPI

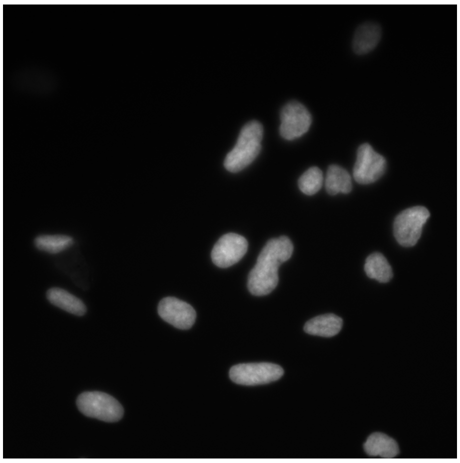

S3B Fig
Nikon Microscope Eclipse 80i

Only Cy3

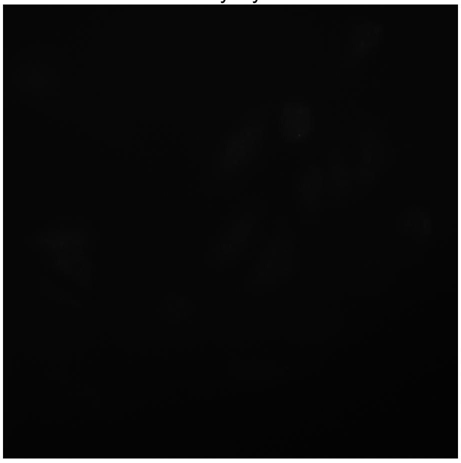

S3B Fig
Nikon Microscope Eclipse 80i
