# Supplementary material for: A universal method for the purification of C2H2 zinc finger arrays
Source: PLoS One. 2025 Feb 4;20(2):e0318295. doi: 10.1371/journal.pone.0318295 (PMC11793764; doi:10.1371/journal.pone.0318295)
Supplement: S1 Table — (DOCX) [file pone.0318295.s005.docx]

**S1 Table. Sequences of ZF proteins**

**CCR5L**

GSLEAAMAERPFQCRICMRNFSDRSNLSRHIRTHTGEKPFACDICGRKFAISSNLNSHTKIHTGSQKPFQCRICMRNFSRSDNLARHIRTHTGEKPFACDICGRKFATSGNLTRHTKIHLRGSGSSLPCTGAA

**CCR5R**

GSLEAAMAERPFQCRICMRNFSRSDNLSVHIRTHTGEKPFACDICGRKFAQKINLQVHTKIHTGEKPFQCRICMRNFSRSDVLSEHIRTHTGEKPFACDICGRKFAQRNHRTTHTKIHLRGSGSSLPCTGAA

**CXCR4L**

GSERPFQCRICMRNFSDRSALSRHIRTHTGEKPFACDICGRKFARSDDLTRHTKIHTGSQKPFQCRICMRNFSQSGNLARHIRTHTGEKPFACDICGRKFAQSGSLTRHTKIHLRGSGSSLPCTGAA

**CXCR4R**

GSERPFQCRICMRNFSRSDSLLRHIRTHTGEKPFACDICGRKFARSDHLTTHTKIHTGSQKPFQCRICMRNFSRSDSLSAHIRTHTGEKPFACDICGRKFADRSNLTRHTKIHLRGSGSSLPCTGAA

**ZVEGF**

GSCGSTGEKPYACPECGKSFSDRSNLTRHQRTHTGEKPYKCPECGKSFSMSHHLSRHQRTHTGEKPYKCPECGKSFSRSDHLSRHQRTHTGRNS

**TZAP_11_**

GSCGSGSGSKGVRKFECTECGYKFTRQAHLRRHMEIHDRVENYNPRQRKLRNLIIED

**TZAP_9-11_**

GSERPFSCEFCEQRFTEKGPLLRHVASRHQEGRPHFCQICGKTFKAVEQLRVHVRRHKGVRKFECTECGYKFTRQAHLRRHMEIHDRVENYNPRQRKLRNLIIEDEKMVVVALQPPAELEVGSAEVIVESLAQGGLASQLPGQRLCAEESFTGPGVLEPSLIITAAVPEDCDT

**ZBrf1**

GSGTERPFACDICGRKFARKYHLQRHTRIHTGEKPFACDICGRKFARRYSLSRHTKIHTQRPQIPPKPFACDICGRKFARKDHLRNHTRIHTGEKPFACDICGRKFARLDVLRAHTKIHTQRPQIPPKPFACDICGRKFAQKSDLGRHTRIHTGEKPFACDICGRKFALKYYLRRHTKIHLRGSCGSPKKKRKV
