# Supplementary material for: A universal method for the purification of C2H2 zinc finger arrays
Source: PLoS One. 2025 Feb 4;20(2):e0318295. doi: 10.1371/journal.pone.0318295 (PMC11793764; doi:10.1371/journal.pone.0318295)
Supplement: S2 Table — (DOCX) [file pone.0318295.s006.docx]

**S2 Table. Efficiencies of fluorophore coupling to ZF proteins.**

| ZFPs | C_ZFPs_ (M) | C_Sulfo-Cy5_ (M) | Labeling efficiency |
| --- | --- | --- | --- |
| CCR5L | 1.5 x 10^-5^ | 7.8 x 10^-6^ | 52.0% |
| CCR5R | 1.6 x 10^-5^ | 1.2 x 10^-5^ | 75.0% |
| CXCR4L | 8.5 x 10^-6^ | 5.6 x 10^-6^ | 65.9% |
| CXCR4R | 1.4 x 10^-5^ | 7.0 x 10^-6^ | 50.0% |
| ZVEGF | 1.5 x 10^-5^ | 1.0 x 10^-5^ | 66.7% |
| TZAP_11_ | 1.4 x 10^-5^ | 1.1 x 10^-6^ | 78.6% |
| TZAP_9-11_ | 1.6 x 10^-5^ | 8.3 x 10^-6^ | 51.2% |
| ZBrf1 | 1.2 x 10^-5^ | 7.6 x 10^-6^ | 63.3% |
