# Supplementary material for: A universal method for the purification of C2H2 zinc finger arrays
Source: PLoS One. 2025 Feb 4;20(2):e0318295. doi: 10.1371/journal.pone.0318295 (PMC11793764; doi:10.1371/journal.pone.0318295)
Supplement: S3 Table — (DOCX) [file pone.0318295.s007.docx]

**S3 Table. Concentrations of fluorescent ZF proteins and DNA oligonucleotides used for MST measurements.**

| ZFPs | DNA | ZFPs concentration  (M) | Range of DNA concentrations  (M) |
| --- | --- | --- | --- |
| CCR5L | ccr5l-wt | 2 x 10^-8^ | 5.0 x10^-6^ ~ 1.5 x 10^-10^ |
|  | ccr5l-mut | 2 x 10^-8^ | 5.0 x10^-6^ ~ 1.5 x 10^-10^ |
| CCR5R | ccr5r-wt | 2 x 10^-8^ | 2.0 x 10^-5^ ~ 6.1 x 10^-10^ |
|  | ccr5l-mut | 2 x 10^-8^ | 5.0 x 10^-4^ ~ 1.5 x 10^-8^ |
| CXCR4L | cxcr4l-wt | 1 x 10^-9^ | 3.1 x 10^-7^ ~ 9.4 x 10^-12^ |
|  | cxcr4l-mut | 1 x 10^-9^ | 3.1 x 10^-7^ ~ 9.4 x 10^-12^ |
| CXCR4R | cxcr4r-wt | 1 x 10^-9^ | 3.1 x 10^-7^ ~ 9.4 x 10^-12^ |
|  | cxcr4r-mut | 2 x 10^-8^ | 5.0 x10^-6^ ~ 1.5 x 10^-10^ |
| ZVEGF | vegf-wt | 2 x 10^-8^ | 5.0 x10^-6^ ~ 1.5 x 10^-10^ |
|  | vegf-mut | 2 x 10^-8^ | 5.0 x10^-6^ ~ 1.5 x 10^-10^ |
| TZAP_11_ | tzap-wt | 2 x 10^-8^ | 1.0 x10^-5^ ~ 3.1 x 10^-10^ |
|  | tzap-mut | 2 x 10^-8^ | 1.0 x10^-5^ ~ 3.1 x 10^-10^ |
| TZAP_9-11_ | tzap-wt | 2 x 10^-8^ | 7.5 x 10^-6^ ~ 2.3 x 10^-10^ |
|  | tzap-mut | 2 x 10^-8^ | 5.0 x 10^-5^ ~ 1.5 x 10^-9^ |
| ZBrf1 | brf1-wt | 2 x 10^-8^ | 5.0 x10^-6^ ~ 1.5 x 10^-10^ |
|  | brf1-mut | 2 x 10^-8^ | 5.0 x10^-6^ ~ 1.5 x 10^-10^ |
