# Supplementary material for: A universal method for the purification of C2H2 zinc finger arrays
Source: PLoS One. 2025 Feb 4;20(2):e0318295. doi: 10.1371/journal.pone.0318295 (PMC11793764; doi:10.1371/journal.pone.0318295)
Supplement: S4 Table — (DOCX) [file pone.0318295.s008.docx]

**S4 Table. DNA binding results of ZFPs.**

| ZFPs | Target DNA | Kd (M) | mut to wt ratio |
| --- | --- | --- | --- |
| CCR5L | ccr5l-wt | 5.7 x 10^-9^ | 131.6 |
|  | ccr5l-mut | 7.5 x 10^-7^ |  |
| CCR5R | ccr5r-wt | 1.6 x 10^-7^ | 61.3 |
|  | ccr5r-mut | 9.8 x 10^-6^ |  |
| CXCR4L | cxcr4l-wt | 3.2 x 10^-10^ | 14.7 |
|  | cxcr4l-mut | 4.7 x 10^-9^ |  |
| CXCR4R | cxcr4r-wt | 1.8 x 10^-10^ | 77.8 |
|  | cxcr4r-mut | 1.4 x 10^-8^ |  |
| ZVEGF | vegf-wt | 1.2 x 10^-8^ | 41.7 |
|  | vegf-mut | 5.0 x 10^-7^ |  |
| ZBrf1 | brf1-wt | 4.1 x 10^-9^ | 1.3 |
|  | brf1-mut | 5.2 x 10^-9^ |  |
| TZAP_11_ | tzap-wt | 1.4 x 10^-7^ | 22.9 |
|  | tzap-mut | 3.2 x 10^-6^ |  |
| TZAP_9-11_ | tzap-wt | 1.7 x 10^-7^ | 34.1 |
|  | tzap-mut | 5.8 x 10^-6^ |  |
