# Supplementary material for: A universal method for the purification of C2H2 zinc finger arrays
Source: PLoS One. 2025 Feb 4;20(2):e0318295. doi: 10.1371/journal.pone.0318295 (PMC11793764; doi:10.1371/journal.pone.0318295)
Supplement: Attachment — Submitted filename: Comments on PONE-D-24-50585.doc [file pone.0318295.s009.doc]

**Review comments**

Liang et.al. reported a universal method for zinc finger protein purification involving two chromatographic steps. As we know, there are different groups of zinc fingers based on the overall shapes pf the protein backbone in the folded domain. Here authors only investigated C2H2 ZF proteins. Does this method apply to other types of protein, such as C4 or C6 ZF proteins? Moreover, purification was the main focus of the studies and the importance of buffer conditions used in purification process was emphasized in the discussion section. However, there is no detailed information in the main text or figures to show this part. Therefore, the manuscript should be revised accordingly before being accepted for publication.

Major points

1. Lack of information related to the optimization of buffer conditions, such as adding L-arginine before or after purification.
2. Lack additional experiments to validate the method to other types of ZF proteins.

Minor points

1. The figures quality can be improved with higher resolution.
2. Line 55, Remove e.g..
3. Line 66-72, “A general method for the purification of ZF…of probes for non-repetitive sequences.” Should move before Line 55.
4. Line 120-121, Wrong place for sentence “IPTG induction led to a prominent band of the expected molecular weight in whole cell lysates (Fig. 1A, compare lanes with and without IPTG).”
5. Fig 1E, the size of the CXCR4L was slightly not consistent in two SDS-PAGE. Why not just simply show the Marker band in the gel too?
6. Line 170, control experiments are needed in Fig.S2. A non-specific probe should be included.
7. Fig.4. is there non-specific binding of TZAP_9-11_ in telomeres (shown in green)? Whether this signal (extra green signal) comes from Cy3 signal?
8. Line 257, remove second “pH7.5”.
9. Line 260 and line 263, the purpose of using two different buffer, 100 mM L-Arg-HCl vs 100 mM L-Arg.
10. Why only show TZAP_9-11_ in Fig. 2B?
11. Information related to yield of each 8 tested proteins can be added to table 1.
